# A bioinformatics approach for identifying the probable cause of the cross-interaction of antibodies to the antigenic protein HPV16 L1 with the HPV6 L1 protein

**DOI:** 10.18699/VJ21.090

**Published:** 2021-11

**Authors:** A.S. Stolbikov, R.K. Salyaev, N.I. Rekoslavskaya

**Affiliations:** Siberian Institute of Plant Physiology and Biochemistry of the Siberian Branch of the Russian Academy of Sciences, Irkutsk, Russia; Irkutsk State University, Irkutsk, Russia; Siberian Institute of Plant Physiology and Biochemistry of the Siberian Branch of the Russian Academy of Sciences, Irkutsk, Russia; Siberian Institute of Plant Physiology and Biochemistry of the Siberian Branch of the Russian Academy of Sciences, Irkutsk, Russia

**Keywords:** human papillomavirus, HPV6 L1, HPV16 L1, bioinformatics analysis, вирус папилломы человека, ВПЧ6 L1, ВПЧ16 L1, биоинформационный анализ

## Abstract

This paper describes an attempt to analyze, with the aid of bioinformatics resources (programs and databases), the probable cause of the cross-interaction of antibodies against HPV16 L1 with antigenic protein HPV6 L1, which has been revealed in the investigation of the candidate vaccine obtained on the base of a plant expression system (tomato plants). In our opinion, the most likely reason for the cross-interaction of antibodies with antigens of different pathogenic HPV types is the similarity of their antigenic determinants. In this work, the amino acid sequences of HPV16 L1 and HPV6 L1 used for the development of a binary vaccine against cervical cancer and anogenital papillomatosis have been analyzed. For the analysis of antigenic determinants, the programs BepiPred-2.0: Sequential B-Cell Epitope Predictor, DiscoTope 2.0 Server and SYFPEITHI have been used. As a result of the analysis of probable B-cell linear determinants (epitopes), it has been found that in both types of HPV the proteins have approximately the same location and size of linear antigenic determinants; the difference is observed only in the form of small shifts in the size of several amino acid residues. However, there are some differences in the amino acid composition of epitopes; therefore, the possibility for cross-interaction of the antibodies with the antigens due to the similarity of linear antigenic determinants for B-cells is very small. The analysis of potential threedimensional epitopes for B-cells has shown that due to little difference between them the HPV16 L1 and HPV6 L1 proteins have no prerequisites for cross-interaction of the antibodies with the antigens belonging to the two different pathogenic HPV types. The analysis of probable linear epitopes for T-cells has revealed a common antigenic determinant in the two protein sequences. According to the rank made with the SYFPEITHI program, the amino acid sequence AQL(I)FNKPYWL is the second most likely antigenic determinant for T-cells. Meanwhile, the amino acid sequences of this determinant in HPV16 L1 and HPV6 L1 are virtually identical. There is a difference in only one position, but it is not critical due to the similarity of the physicochemical properties of amino acids, for which there is a replacement in the amino acid sequence of antigenic determinants. Consequently, some moderate cross-interaction of the antibodies to HPV16 L1 with the antigens of HPV6 L1 may be expected.

## Abstract (ru)

C помощью биоинформационных ресурсов (программ и баз данных) предпринята попытка проанализировать вероятную причину перекрестного взаимодействия антител против ВПЧ16 L1 с антигенными белками ВПЧ6 L1, которое было выявлено при изучении кандидатной вакцины, полученной на основе растительной экспрессионной системы (растений томата). По нашему мнению, наиболее вероятной причиной перекрестного взаимодействия антител с антигенами, принадлежащими к разным патогенным типам вируса папилломы человека (ВПЧ), является сходство антигенных детерминант. В ходе исследования были проанализированы аминокислотные последовательности ВПЧ16 L1 и ВПЧ6 L1, которые использовались при разработке бинарной вакцины против цервикального рака и аногенитальных папилломатозов. Для анализа антигенных детерминант использовались программы BepiPred-2.0: Sequential B-Cell Epitope Predictor, DiscoTope 2.0 Server, SYFPEITHI. В результате исследования вероятных линейных детерминант для В-клеток установили, что у обоих типов ВПЧ белки имеют примерно одинаковое расположение и размер линейных антигенных детерминант, отличие наблюдается только в виде небольших сдвигов в несколько аминокислотных остатков. Однако выявлено некоторое различие в аминокислотном составе эпитопов, поэтому потенциал перекрестного взаимодействия антител с антигенами за счет сходства линейных антигенных детерминант для В-клеток незначителен. Анализ потенциальных трехмерных эпитопов для В-клеток показал, что по сумме различий белки ВПЧ16 L1 и ВПЧ6 L1 не имеют предпосылок для перекрестного взаимодействия антител с антигенами, принадлежащими к двум разным патогенным типам ВПЧ. Анализ вероятных линейных эпитопов для Т-клеток обнаружил у двух белковых последовательностей общую антигенную детерминанту. Согласно рейтингу, составленному программой SYFPEITHI, аминокислотная последовательность AQL(I)FNKPYWL представляет собой вторую, по вероятности, антигенную детерминанту для Т-клеток. При этом аминокислотная последовательность данной детерминанты у ВПЧ16 L1 и ВПЧ6 L1 практически идентична. Отличие имеется лишь по одной позиции, но оно не является критичным в силу сходства физико-химических свойств аминокислот, по которым наблюдается замена в аминокислотной последовательности антигенных детерминант. Исходя из этого можно ожидать умеренно выраженное перекрестное взаимодействие антител к ВПЧ16 L1 с антигенами ВПЧ6 L1.

## Introduction

Tens of millions of people are infected every year with various types of human papillomavirus (HPV), and this accounts only for regions of the world where appropriate medical observations and statistics are conducted (McLaughlin-Drubin, Münger, 2009). Therefore, the development of preventive vaccines against HPV is one of the current challenges to curb the increase in the number of diseases caused by this type of infectious agents.

The development of candidate vaccines based on plant expression systems is a relatively new field of biofarming. Plant expression systems have certain advantages over other systems. First of all, these advantages are related to safety due to the absence of prions, mammalian pathogens, transposons and dangerous viruses in a latent state, as well as the relative cheapness of obtaining vaccines, which generally contributes to wider commercialization and scaling. In our previous investigation, we attempted to develop candidate tetravalent oral vaccine based on transgenic plants against four types of HPV (16, 18, 31, 45) capable of causing cervical cancer. In this work, we planned to develop a vaccine that would provide maximum protection against cervical cancer by using the main antigenic protein L1 of the viral envelope of four highly oncogenic types of human papillomaviruses (HPV16, HPV18, HPV31 and HPV45), which are responsible for most cases of cervical cancer.

It has been revealed that the antibodies to the antigenic protein HPV16 L1 successfully interact with the HPV18 L1, HPV31 L1 and HPV45 L1 antigens (Salyaev et al., 2017). Based on the data obtained, it was assumed that the crossinteraction of the antibodies with the antigens belonging to different pathogenic types of HPV may be due to the similarity of antigenic determinants. This assumption was verified with a bioinformatic approach, where common linear determinants for T cells and B cells were found in all four types of L1 viral proteins. In addition, similar three-dimensional antigenic determinants were found for B cells in HPV16 L1 and HPV18 L1 (Stolbikov et al., 2020). When working on the binary vaccine containing HPV16 L1 and HPV6 L1 antigenic proteins, Western blot hybridization revealed a cross-interaction of serum antibodies against HPV16 L1 with antigenic protein HPV6 L1 (Salyaev et al., 2017; Rekoslavskaya et al., 2021). Human papillomavirus type 6 does not cause cancer, but can lead to the development of anogenital and respiratory papillomatoses. Despite the fact that these diseases rarely lead to death, they are widespread and highly contagious (WHO, January 11, 2020).

Such a wide range of cross-interaction between antigens and antibodies, which goes beyond the viruses that cause cervical cancer and belong to another family, seemed extremely interesting to us. In this regard, in this work, the antigenic determinants of HPV16 L1 and HPV6 L1 have been subjected to a comparative bioinformatic analysis. The data obtained during this work can be used to optimize the development of candidate vaccines using fewer HPV types due to the crossinteraction between antibodies and antigens of unrelated types, which, in turn, will reduce the labor intensity and cost of production of vaccines against dangerous types of human papillomaviruses.

## Materials and methods

Alignment of the amino acid sequences of HPV16 L1 and HPV6 L1. As the first stage of the analysis of the antigenic determinants of HPV16 L1 and HPV6 L1, paired alignment of HPV isolates of each type was conducted. For this purpose, their full-size amino acid sequences encoded by nucleotide sequences previously used in genetic constructs in the development of the binary vaccine against cervical cancer and anogenital papillomatosis were found and processed in the NCBI database (GenBank) (Salyaev et al., 2017). This was necessary for the subsequent determination of the difference in the antigenic determinants of the two types of HPV. Whole set of full-size amino acid sequences of HPV16 L1 and HPV6 L1 was also extracted from the GenBank database. The alignment of amino acid sequences was carried out using the editor of multiple alignment of nucleotide and amino acid sequences BioEdit. The phylogenetic tree was constructed using the program “Simple Phylogeny” (EMBL-EBI) by Nearest Neighbor Algorithms (the neighbor-joining method) and unweighted pairwise mean (UPGMA).

Identification of potential antigenic determinants. For the second stage of the analysis of antigenic determinants, the program “BepiPred-2.0: Sequential B-Cell Epitope Predictor” was used (http://www.cbs.dtu.dk/services/BepiPred/(Jespersen et al., 2017). This bioinformatic resource allowed us to identify potential linear antigenic determinants for B cells.

To determine the three-dimensional antigenic determinants for B cells, the program “DiscoTope 2.0 Server” was used (http://www.cbs.dtu.dk/services/DiscoTope/) (Kringelum et al., 2012). When working with this program, the strictest conditions were set: sensitivity 47 %, specificity 75 %. Threedimensional models of proteins that were analyzed using the program DiscoTope 2.0 Server were found in the Protein Data Bank (PDB) database. The programs BepiPred-2.0: Sequential B-Cell Epitope Predictor and DiscoTope 2.0 Server were publicly available on the server of the Danish Technical University (DTU). The search for potential antigenic determinants for T cells was performed using the SYFPEITHI program, which is in the public domain http://www.syfpeithi.com. This bionformatic resource ranks all possible variants of antigenic determinants according to the probability of their interaction with T cells (Rammensee et al., 1999).

## Results

Amino acid alignment

**Fig. 1.**
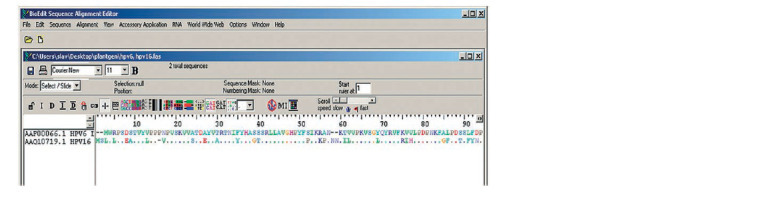
The alignment of amino acid sequences of HPV6 L1 and HPV16 L1 in the BioEdit program.

The amino acid sequences of the L1 capsid proteins of HPV16 and HPV6 viruses were downloaded from the NCBI international database and aligned in the BioEdit program (Fig. 1).

**Fig. 2.**
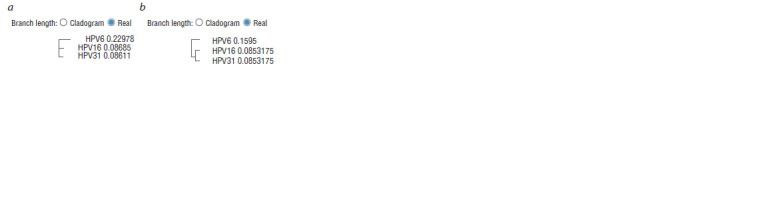
The phylograms constructed using the program Simple Phylogeny (EMBL-EBI) by the neighbor-joining method (a) or by the UPGMA method (b).

According to the results of the alignment, phylograms were built. To emphasize the evolutionary differences between the 6 and 16 types of HPV, the comparative analysis used HPV31, which belongs to the same species Alphapapillomavirus 9 as HPV16 (Fig. 2). The phylogenetic comparison data showed significant differences between HPV6 L1 and HPV16 L1.

Analysis of linear antigenic determinants for B cells

**Fig. 3.**
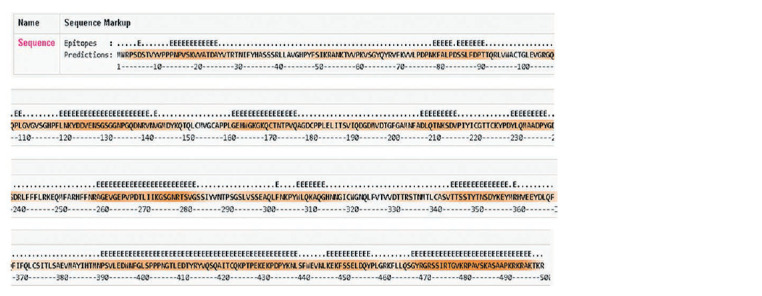
Probable antigenic determinants of protein HPV6 L1 (BepiPred-2.0: Sequential B-Cell Epitope Predictor). Epitope Threshold = 0.5; E – antigenic determinants.

The amino acid sequences of two viral proteins HPV6 L1 and HPV16 L1 were analyzed for the presence of potential linear antigenic determinants for B cells using the program BepiPred-2.0: Sequential B-Cell Epitope Predictor. The study showed that the HPV6 L1 protein has the following antigenic determinants: 14–25, 79–83, 85–91, 120–141, 162–177, 208–216, 230–238, 260–283, 308–314, 345–358, 391–439, 447–457, 468–497 (Fig. 3).

Previously, the following antigenic determinants were identified in HPV16 L1: 8–28, 83–95, 123–143, 166–177, 213–220, 234–243, 264–286, 350–369, 396–421, 426–444, 452–462, 473–502 (Stolbikov et al., 2020).

As one can see, the analysis of linear determinants gave evidence that in the HPV types studied, the proteins have approximately the same location and size of linear antigenic determinants, the difference is observed only in the form of small shifts in several amino acid residues. A substantial difference consists only in the presence of linear determinants in the HPV6 L1 protein: 79–83, 308–314, which are not observed in HPV16 L1. In addition, the antigenic determinant 426–444 is present in the HPV type 16 protein, which has not been revealed in the virus type 6 protein.

In order to draw the conclusion that there are similar linear antigenic determinants in the two HPV types under consideration, it was necessary to compare the amino acid composition of the proposed epitopes. The difference in the amino acid composition can lead to a decrease in the degree of affinity with antibodies. The absence of substitutions in amino acid sequences or substitutions with amino acids similar in properties can preserve the level of antibody affinity. Therefore, it is important to determine the presence and evaluate the quality of amino acid substitutions in the proposed epitopes of the HPV6 L1 and HPV16 L1 proteins.

**Fig. 4.**
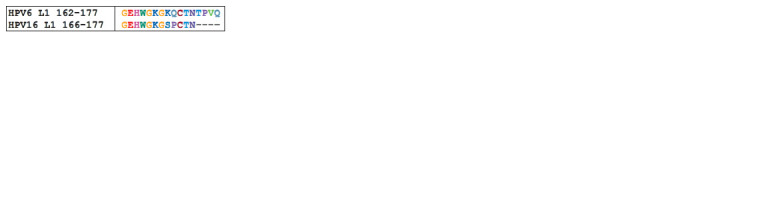
The result of amino acid alignment of presumed antigenic determinant 166–177

The paired alignment of protein sequences demonstrated substantial difference in the amino acid composition in most of the antigenic determinants. However, definite similarity was found between some amino acid sequences located within the boundaries of antigenic determinants close to both proteins. For eхample, in the determinant 166–177 for HPV16 L1, there was a difference in two amino acids: lysine was replaced with serine, glutamine with proline (Fig. 4). Serine is a polar uncharged oxycarboxylic amino acid, and lysine is a polar positively charged one. Proline is a heterocyclic nonpolar amino acid, and glutamine is a polar uncharged one. Taking into account the difference in the physico-chemical properties of amino acids, for which there are differences in the supposed antigenic determinants, as well as the difference in the length of linear epitopes, we may assume a weak cross-interaction with antibodies.

**Fig. 5.**
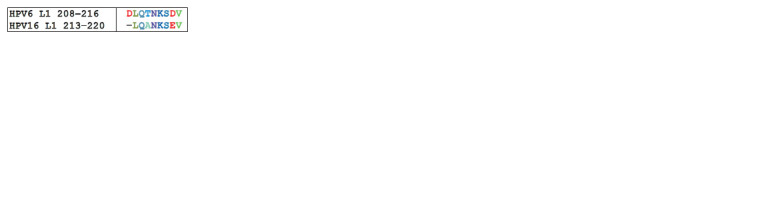
The result of amino acid alignment of the presumed antigenic determinant 213–220.

In the determinant 213–220 for HPV16 L1, some difference of two amino acids was found to be relative to the HPV6 L1 sequence: threonine was replaced with alanine, and aspartic acid was replaced with glutamic acid (Fig. 5). Since threonine substantially differs from alanine in physical and chemical properties, such a replacement can affect the antigenic properties of the L1 protein. In case of the second substitution, we deal with polar negatively charged amino acids with almost identical physical and chemical properties. Considering this fact, as well as the fact that the amino acid substitutions in the sequences are located at some distance from each other,we can assume that there are similar antigenic properties of this region for the two viral proteins 

Search for antigenic determinants for T cells

In order to obtain additional evidence of influence of the similarity of the antigenic determinants of viral proteins HPV6 L1 and HPV16 L1 on the cross-interaction of the antibodies with the antigens, which belong to the two pathogenic types of human papillomavirus, a search for potential antigenic determinants for T cells was conducted with the aid of the bioinformatic resource SYFPEITHI. This program ranks possible variants of antigenic determinants according to the probability of their interaction with T cells.

**Fig. 6.**
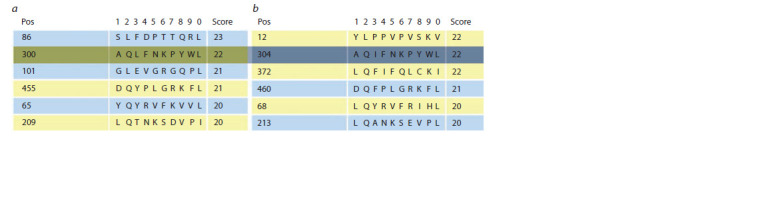
Probable antigenic determinants for T cells (HLA-B13 decamers) in the protein sequences HPV6 L1 (a) and HPV16 L1 (b) according to program SYFPEITHI.

Analysis of the protein sequences of the two scrutinized viral proteins allowed us to reveal a common probable antigenic determinant for T cells, which was located in HPV6 L1 at position 300–309, and in HPV16 L1 at position 304–313 of the amino acid sequence. According to the rating compiled by program SYFPEITHI, the amino acid sequence AQL(I) FNKPYWL was the second most likely antigenic determinant for T cells (Fig. 6).

When comparing the amino acid sequence of this determinant, a difference of only one amino acid was revealed in the two proteins, in HPV16 L1 leucine was replaced with isoleucine. Such a replacement should not lead to a change in the antigenic properties of the determinant, since leucine and isoleucine belong to the same class of amino acids and have similar physico-chemical properties. As a result, it should be noted that this antigenic determinant in the HPV6 L1 and HPV16 L1 proteins may have similar antigenic properties. Furthermore, multiple alignment of all the full-length amino acid sequences of HPV16 L1 and HPV6 L1 presented in NCBI has shown that this segment of the sequence is probably highly conserved and is almost identical in these two proteins.

Investigation of potential three-dimensional epitopes for B cells

Only one three-dimensional model of the HPV6 L1 protein was found in the PDB database (6L31, DOI 10.2210/pdb6l31/ pdb). Unfortunately, this model was not informative for the program DiscoTope 2.0 Server, so we conducted a comparative analysis of three-dimensional antigenic determinants using literary data.

According to some scientific publications, the following domains are isolated from the HPV6 L1 protein, forming threedimensional epitopes that can interact with B cells: F49, R53, A54; K52, R53, A54, N55; Y123, N128; G130, S131, G132; K169, T172, N173, P175, V176, Q177, A178; E262, V263, E265, P266; V344, T345, T346; S353. Critical paratopes for recognition are domains F49, R53, A54 and K169, T172, N173, P175, V176, Q177, A178 (McClements et al., 2001).

It was shown in our previous publication that the HPV16 L1 protein has a spatial epitope in domain K53–L61, which partially coincides with the critical domain F49, R53, A54 of the HPV6 L1 protein. In addition, the location of the epitope S353 of the HPV6 L1 protein coincides with the three-dimensional antigenic determinant T350–Y355 of the HPV16 L1 protein (Stolbikov et al., 2020). In order to determine the level of similarity of the immunological properties of these two proteins, we analyzed the paired alignment of the amino acid sequences in the domains of their assumed three-dimensional antigenic determinants. Inconsistencies in amino acid residues were found in the supposed epitopes of antigenic proteins. In the position of 53 amino acid sequence in HPV16 L1, arginine is replaced with lysine, and in the position of 353 – serine with glutamic acid. These amino acid substitutions may be considered insignificant due to the similarity of the physicochemical characteristics of the corresponding amino acids.

## Discussion

According to the results obtained by us, it can be stated that there is a definite similarity between the antigenic determinants of HPV16 L1 and HPV6 L1 proteins. At the same time, with regard to B cells, the potential for cross-interaction of antibodies with antigens due to the similarity of linear antigenic determinants and three-dimensional epitopes is not substantial. However, for two types (16 and 6) of L1 viral proteins, there is a substantial similarity of linear antigenic determinants for T cells. According to the results obtained with the aid of program SYFPEITHI, these determinants are in the second position, but, despite this, these have a fairly high probability score. At the same time, the amino acid sequences of these epitopes are almost identical. There is some difference only in one position, but it is not critical due to the similarity of the physico-chemical properties of amino acids, according to which there is a replacement of antigenic determinants in the amino acid sequence. Based on the above results, when immunizing HPV16 L1, we can expect a fairly reasonable cross-interaction of antibodies with HPV6 L1 antigens.

The results obtained give a definite explanation of the cause of the effect of cross-interaction of antibodies with antigens belonging to different pathogenic types of HPV identified earlier. However, in order to obtain a more complete understanding of the mechanism of cross-interaction, it is desirable to study the phenomenon of polymorphic distribution of epitopes and the process of induction of de novo antibody synthesis (Brown et al., 2009; Kemp et al., 2011; Scherpenisse et al., 2013; Nakagawa et al., 2015).

## Conclusion

The efficiency and expediency of the approaches and methods used in this work is confirmed by publications of other scientists. For example, it is known from the literary sources that a team of authors (Namvar et al., 2019) have used the same bioinformatic resources as our team (BepiPred-2, SYFPEITHI) to conduct an investigation of the cross-immune response to the surface proteins L1 and L2 of human papillomaviruses of highly oncogenic types 16 and 18. In this work, much attention was paid to the comparison of amino acid properties, such as hydrophobicity, the surface area accessible to the solvent, the charge and the secondary structure of the identified similar antigenic determinants of two different types of HPV. As a result of this investigation, a candidate multiepitope vaccine was created, the tests of which on laboratory mice showed rather good results. Application of this recombinant vaccine helped to induce a sufficiently strong immune response and protected mice from tumor cells with an efficiency of about 66.67 % (Namvar et al., 2019).

In conclusion, it should be noted that application of the research methods discussed above can substantially accelerate the development of efficient broad-spectrum vaccines against highly dangerous types of HPV. To date, there is a vaccine ‘Gardasil-9’ that provides protection against 9 types of oncogenic HPV, but it already contains the maximum permissible amount of antigenic proteins (270 μg of protein in one dose), while it does not provide protection in about 10 % of cases (Li et al., 2018). Therefore, an extended study of cross-interaction of antibodies with antigens belonging to different pathogenic types of HPV conducted with the aid of bioinformatic analysis techniques can help in development of multi-epitope wideaction vaccines without increasing the number of antigenic proteins in the vaccine preparations.

## Conflict of interest

The authors declare no conflict of interest.
